# Anatomical Variations in Permanent Mandibular Molars in the Sharjah Population: A Cohort Study Using Cone‐Beam Computed Tomography

**DOI:** 10.1155/ijod/5613749

**Published:** 2026-04-30

**Authors:** Shaima Dheyab, Entisar AlRasasi, Ahmed M. Aziz, Saad Albayatti, Mehmet Omer Gorduysus

**Affiliations:** ^1^ Department of Restorative Dentistry, College of Dental Medicine, University of Sharjah, Sharjah, UAE, sharjah.ac.ae; ^2^ Department of Dental Services, Dubai Health Authority, Dubai, UAE, dha.gov.ae; ^3^ Department of Oral and Craniofacial Health Sciences, College of Dental Medicine, University of Sharjah, Sharjah, UAE, sharjah.ac.ae

**Keywords:** anatomical variations, cone-beam computed tomography, mandibular molar, root canal morphology

## Abstract

**Objective:**

This cohort study aimed to assess the prevalence of anatomical variations, including radix entomolaris (RE), radix paramolaris (RP), middle mesial canal (MMC), and C‐shaped canal, in the permanent mandibular first and second molars across different nationalities and genders.

**Materials and Methods:**

A total of 777 cone‐beam computed tomography scans were retrospectively evaluated from patients receiving routine dental treatment at the University Dental Hospital, Sharjah, United Arab Emirates (UAE), of which 165 met the inclusion criteria. Descriptive statistics were presented as the overall number of canals and the prevalence and distribution of RE, RP, MMC, and C‐shaped canals in the permanent mandibular first and second molars. Chi‐square tests were performed for comparative analyses.

**Results:**

None of the mandibular first molars included had RE, RP, or MMC. No gender differences were detected. One participant (0.30%) had a C‐shaped canal configuration. In mandibular second molars, the overall prevalence of C‐shaped canal anatomy was 4.54%, and 0.30% for RE. A case of RE was exclusively observed in one first molar, with no instances of RP or MMC identified in our study population.

**Conclusion:**

In this study, the majority of permanent first molars exhibited two roots and four canals, while permanent second molars most frequently had two roots and three canals, with greater variations revealed in second molars and most frequently observed in Pakistani patients. A C‐shaped canal configuration was more common in the second molars than in the first ones. Overall, Asian and African ethnicities tended to have significantly more canal variations than Western populations.

**Clinical Significance:**

The clinical relevance of this study lies in its potential to enhance diagnostic accuracy, treatment planning, and patient outcomes by understanding common variations and their prevalence across various ethnic groups. Therefore, clinicians can better anticipate the challenges and implications of treatment.

## 1. Introduction

One of the primary objectives of endodontic treatment is to eradicate bacteria and microorganisms from the root canal system. The successful elimination of these microorganisms ensures comprehensive endodontic treatment and subsequently improves prognostic outcomes. Its main principle relies on a comprehensive understanding of root canal anatomy and morphology, including the identification of unusual anatomical variations and subsequent preparation of the canal. Failure to detect and treat undetected canals increases the risk of persistent infection and subsequent treatment failures.

The mandibular first and second molars typically have two roots. A mesial root with typically two canals, mesiobuccal and mesiolingual, and a distal root that has either one or two canals [1–3]. However, anatomical variations have been observed across diverse ethnic groups and even among patients within the same ethnic background [4].

Despite their typical two‐rooted configuration, mandibular molars may present with several anatomical variations including radix entomolaris (RE), radix paramolaris (RP), middle mesial canal (MMC), and C‐shaped canals. RE typically involves a third root on the distolingual surface of the first and second mandibular molars. It is often superimposed by the distal root on preoperative radiographs, making its detection difficult. RE often exhibits a pronounced curvature, which can complicate instrumentation and treatment [5].

Carlsen and Alexandersen [6] identified four types of RE: Type A, the cervical part of the RE is located distally and separate from a distal root; Type B, the cervical part of the RE is located distally and connects to a distal root; Type C, the cervical part of the RE is mesially located; and Type AC, the cervical part of the RE is located centrally between the mesial and distal roots.

Meanwhile, RP represents a third root located mesiobuccally. RP is less frequently documented than RE [7], with a reported incidence of 0.3% in a Saudi population [8]. Other variations include MMC, which is a deep canal situated between the developmental groove of the mesiobuccal and mesiolingual canals of the mandibular first and second molars [9].

C‐shaped canals appear as multiple C‐shaped interconnected canals rather than separate ones and are reported most frequently in mandibular second molars [10]. C‐shaped canal anatomy can be identified preoperatively by the presence of a fused or conical root. A C‐shaped canal features a continuous, ribbon‐like or groove‐shaped opening along the floor of the chamber upon accessing the pulp chamber. Fan et al. [11] classified C‐shaped canals into five groups: C1, uninterrupted C‐shaped canal with no separation; C2, discontinued semicolon; C3, two or three separate canals; C4, one round canal; and C5, no canal.

The exact cause of these variations remains unclear, they can be attributed to either genetic or external factors [12]. Although there are no conclusive data on these factors, their frequency has been shown to vary significantly according to geographic region [13]. The presence of additional roots can often be clinically detected by distinctive crown features, such as a bulbous crown outline, a prominent distolingual lobe, or an additional cusp [14]. However, a definitive diagnosis is made based on radiographic examinations and intraoperative findings. High‐quality radiographs that provide precise images are invaluable diagnostic tools for determining the number of roots, canals, and canal curvatures. Cone‐Beam Computed Tomography (CBCT) has proven to be one of the most accurate methods for detecting these variations [15–18]. CBCT offers several advantages over conventional radiography, including superior resolution and accurate interpretation [19]. Nevertheless, CBCT is associated with high radiation and cost [20], which complicates its implementation in some cases.

The existing studies report varying prevalence rates of anatomical variations [21–26]; however, they often fail to critically explore the underlying reasons for these discrepancies. Factors such as population ethnicity, diagnostic methods, tooth type and location, sample size, and statistical comparison with previous data are frequently inconsistent across studies and rarely discussed in detail. Without addressing these issues, conclusions about prevalence remain limited and may lead to overgeneralization of findings. Considering the limitations of existing knowledge, we aimed to assess the prevalence of the number of roots, canals, and anatomical variations, namely RE, RP, MMC, and C‐shaped canal anatomy, in permanent mandibular first and second molars across different ethnic regions and nationalities.

## 2. Materials and Methods

Ethical approval was obtained from the Research Ethics Committee of the University of Sharjah, Sharjah, UAE (REC‐22‐04‐15‐S). A retrospective review of CBCT scans of patients with dental records was conducted at the University Dental Hospital, Sharjah (UDHS). These scans were primarily acquired for diagnosis and treatment planning of various conditions, such as surgically impacted teeth, implant placement, orthodontic procedures, and both surgical and nonsurgical root canal treatments. The axial, sagittal, and coronal planes were viewed in CBCT images. A total of 777 CBCT scans were examined. Scans that met the inclusion and exclusion criteria were included in the analysis. The inclusion criteria were as follows: bilateral presence of permanent mandibular first and second molars, complete root formation, and closed apices of fully erupted teeth. The exclusion criteria were as follows: teeth covered by a fixed prosthesis; those with previous endodontic treatment; teeth with severe calcification in root canals or root resorption seen radiologically as translucencies or irregular root contours; distorted CBCT images; partially formed roots; and open apices. Two examiners were calibrated prior to data collection to ensure consistency in radiographic interpretation. The calibration process consisted of joint training sessions during which a predefined set of reference radiographs representing different root and canal configurations of mandibular molars was independently evaluated and subsequently discussed. Disagreements were resolved through consultation with a third senior reviewer until consensus was achieved. Inter‐ and intra‐examiner reliability were assessed using Cohen’s kappa coefficient. A κ value > 0.80 was considered indicative of almost perfect agreement.

Bilateral presence of mandibular first and second molars; number of roots and canals; as well as the presence of RE, RP, MMC, or C‐shaped canal configuration. Additionally, the patients with detected variations were stratified according to nationality and ethnicity, and a comparative analysis was performed to evaluate the association of these parameters with root and canal morphology.

Data were analyzed using IBM SPSS software (Version 27). Descriptive statistics, including frequencies and percentages, were used to summarize the distribution of root numbers and canal configuration probabilities, as well as the prevalence of morphological variation. The chi‐square test was performed to compare female and male patients, as well as the right and left sides, across all tested variations. Statistical significance was determined at a *p*‐value threshold of 0.05.

## 3. Results

A total of 165 CBCT scans met the inclusion criteria. The enrolled sample consisted of 100 (60.6%) male and 65 (39.4%) female patients. A total of 30 nationalities participated in the study (Table 1). Given the diversity of the sample and the limited number of patients per nationality, nationality‐related findings were reported only for the teeth exhibiting anatomical variations. Nationalities were aligned with their broadly conforming ethnic regions to investigate potential associations with root canal anatomy, and these ethnic regions were designated as ethnicities. This approach also aimed to exclude potential genetic variability caused by including individuals of diverse ethnic origins within one nationality. The most commonly encountered ethnicity was Middle Eastern (*n* = 71%, 43%), followed by South Asian (*n* = 43%, 26%), North African (*n* = 32%, 19.37%), Sub‐Saharan African (*n* = 8%, 4.83%), Western (*n* = 5%, 3.01%), Southeast Asian (*n* = 4%, 2.41%), and East Asian and Latin American (*n* = 1%, 0.60%). The chi‐square test revealed no statistically significant differences across all comparisons (*p* > 0.05).

**Table 1:** Nationalities and ethnicities.

Country	Frequency	Ethnicity
Afghanistan	2 (1.21%)	South Asian
Bangladesh	8 (4.80%)	
India	4 (2.42%)	
Pakistani	29 (17.57%)	
Total	43 (26%)	

Algeria	2 (1.21%)	North African
Egyptian	23 (13.93%)	
Morocco	2 (1.21%)	
Tunisia	1 (0.60%)	
Sudanese	4 (2.42%)	
Total	32 (19.37%)	

Palestine	2 (1.21%)	Middle Eastern
Syrian	14 (8.48%)	
Lebanon	2 (1.21%)	
Iraq	2 (1.21%)	
Iran	2 (1.21%)	
Jordanian	15 (9.09%)	
Kuwait	2 (1.21%)	
UAE	26 (15.75%)	
Yemeni	6 (3.63%)	
Total	71 (43%)	

Indonesia	1 (0.60%)	Southeast Asian
Philippines	3 (1.81%)	
Total	4 (2.41%)	

Nigeria	5 (3.03%)	Sub‐Saharan African
South Africa	1 (0.60%)	
Kenya	1 (0.60%)	
Tanzania	1 (0.60%)	
Total	8 (4.83%)	

South Korea	1 (0.60%)	East Asian

Australian	1 (0.60%)	Western
Canada	1 (0.60%)	
United Kingdom	1 (0.60%)	
American	2 (1.21%)	
Total	5 (3.01%)	

Belize	1 (0.60%)	Latin American

Total	165 (100%)	

### 3.1. Mandibular First Molars

A total of 330 bilateral mandibular first molars were evaluated (Table 2). Of these, 210 (63.63%) had two roots and four canals, distributed as two canals in the mesial root and two canals in the distal root. The prevalence of four canals was higher on the right side, 107 (32.42%), than on the left side, 103 (31.12%). Moreover, mandibular first molars with two roots and three canals (two canals in the mesial root and one in the distal root) were less commonly observed than those with a four‐canal configuration, 119 (36.06%). This configuration was found more frequently on the left side, 61 (18.48%), than on the right side, 58 (17.57%). Only one participant from Southeast Asia exhibited a C‐shaped canal configuration in a mandibular left first molar tooth, accounting for 0.30% of the cases. No instances of MMC, RE, or RP were identified in the mandibular first molars on the analyzed scans.

**Table 2:** Percentages of mandibular first molars with four‐, three‐, and two‐canal C‐shaped canal configurations, radix entomolaris and paramolaris, and middle mesial canals.

Mandibular first molar 330 (100%)		
Two‐rooted (four canals)	210 (63.63%)	
	Right 107 (32.42%)	Left 103 (31.21%)
Two‐rooted (three canals)	119 (36.06%)	
	Right 58 (17.57%)	Left 61 (18.48%)
C‐shaped canal configuration	Right 0 (0.00%)	Left 1 (0.30%)
Radix entomolaris	0 (0.00%)	
Radix paramolaris	0 (0.00%)	
Middle mesial canal	0 (0.00%)	

### 3.2. Mandibular Second Molars

A total of 330 bilateral second molars were assessed (Table 3). Among these, 267 (80.90%) exhibited a configuration of two roots and three canals, comprising two mesial canals and one distal canal. This configuration was more frequent on the right side 135 (40.90%) than on the left side 132 (40%). In contrast, mandibular second molars with two roots and four canals (two canals in the mesial root and two in the distal one) accounted for 44 (13.33%), with 22 (6.66%) on each side. The highest prevalence of second molars with four canals was attributed to South Asian patients, particularly those of Pakistani nationality. Additionally, three (0.90%) of the mandibular second molars had two roots and two canals, with one canal in the mesial root and one in the distal root, with two (0.60%) on the left side and one (0.30%) on the right side. Mandibular second molars with a two‐canal configuration were observed in one male and one female patient from South Asia.

**Table 3:** Percentages of mandibular second molars with four‐, three‐, and two‐canal C‐shaped canal configurations, radix entomolaris and paramolaris, and middle mesial canals.

Mandibular second molar 330 (100%)		
Two‐rooted (four canals)	44 (13.33%)	
	Right 22 (6.66%)	Left 22 (6.66%)
Two‐rooted (three canals)	267 (80.90%)	
	Right 135 (40.90%)	Left 132 (40.00%)
Two‐rooted (two canals)	3 (0.90%)	
	Right 1 (0.30%)	Left 2 (0.60%)
C‐shaped canal configuration	15 (4.54%)	
	Right 7 (2.12%)	Left 8 (2.42%)
Radix entomolaris	Right 0 (0.00%)	Left 1 (0.30%)
Radix paramolaris	0 (0.00%)	
Middle mesial canal	0 (0.00%)	

**Figure 1:**
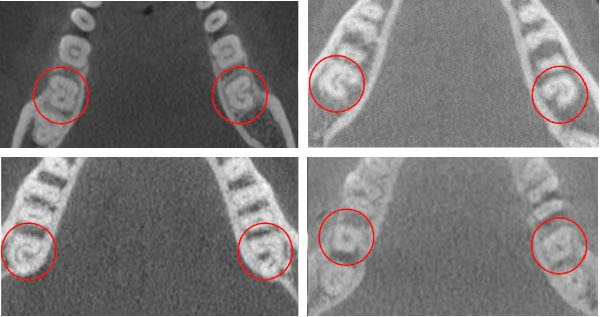
C‐shaped canal anatomy.

**Figure 2:**
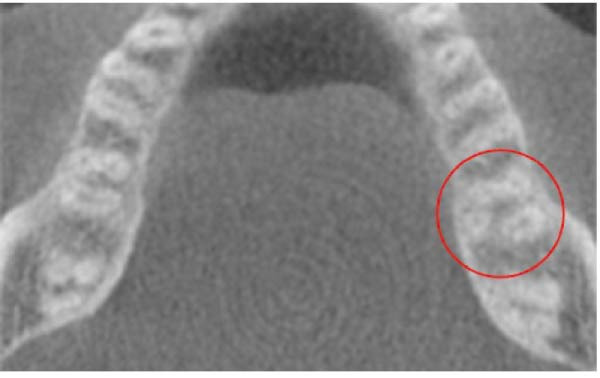
Radix entomolaris in a mandibular second molar tooth.

Moreover, 15 (4.54%) of the evaluated mandibular second molars displayed a C‐shaped root canal configuration (Figure 1), with a slightly greater occurrence on the left side, 8 (2.42%), than on the right side, 7 (2.12%). Table 4 summarizes the findings on the C‐shaped canal. Additionally, one male North African patient presented with RE on the left side (0.30%) (Figure 2), and no MMC or RP were observed in the mandibular second molars.

**Table 4:** Mandibular second molar with C‐shaped canal configuration.

Gender	Ethnicity	Distribution
Female	South Asian	Right and left
Male	South Asian	Right and left
Male	South Asian	Right and left
Male	Middle Eastern	Right and left
Female	Middle Eastern	Right
Male	Middle Eastern	Right and left
Male	North African	Left
Female	North African	Right and left
Male	European	Left

## 4. Discussion

This study aimed to assess the prevalence of the number of roots, canals, and anatomical variations, namely RE, RP, MMC, and C‐shaped canal anatomy, in permanent mandibular first and second molars across different ethnic regions and nationalities, using CBCT scans. The results showed that the two‐root configuration was the most prevalent among the first and second molars, with 643 cases (96.05%). Teeth exhibiting C‐shaped root anatomy were present in 16 cases (2.42%), and this configuration was most commonly detected in the second molars. Only one tooth featured a third distolingual root, comprising 0.15% of the sample. A significantly more pronounced variety of root canal configurations was also revealed.

### 4.1. Four‐Canal Configuration

The analysis of our outcomes indicated that the overall prevalence of mandibular first molars with four canals was 63.63%. Comparable prevalences reported in the literature include 59% in a Sudanese population [27], 46% in a Taiwanese Chinese population [28], 45.8% in Jordanian populations [29], 25.3% in Saudi Arabians [26], and 15.4% in Turkish populations [30]. In contrast, the prevalence of the four‐canal configuration in mandibular second molars was 13.33% in the present study, which significantly differs from previous studies, which have demonstrated varying prevalence rates of 34% in an Iranian population [31], 16.6% in a Jordanian population [29], 2.27% in a Turkish population [30], and 0.9% in an Egyptian population [32]. Clinically, the distal canal is usually located centrally within the distal root, which is an important consideration during access cavity preparation and canal negotiation. However, when two distal canals are present, the distal orifice often appears elongated in the buccolingual direction rather than round. Careful inspection of the pulpal floor for developmental grooves may also aid in detecting an additional canal, which is commonly located either buccally or lingually relative to the main distal canal. This highlights the importance of adequate deroofing and controlled troughing to enable full identification of the distal root canal system.

### 4.2. Three‐Canal Configuration

Mandibular first molars with three canals (two mesial and one distal) were encountered less frequently than those with four canals, with an overall prevalence of 36.06%. Meanwhile, 79.9% of the Turkish population [30], 73% of the Saudi Arabian population [26], 57.73% of the Indian population [33], 51% of the Taiwanese Chinese population [28], and 48.2% of the Jordanian population [29] exhibited this configuration. The three‐canal configuration in mandibular second molars was reported as 80.9% in our study compared to 80% in Egyptians [32], 72.8% in Turks [30], 58.3% in Jordanians [29], and 54% in Iranians [31]. Overall, this configuration was found to be significantly more characteristic of mandibular second molars than first molars.

### 4.3. Two‐Canal Configuration

None of the analyzed mandibular first molars had a two‐canal configuration (two roots and two canals; one mesial and one distal). In contrast, three (0.90%) mandibular second molars had this configuration. According to previously published literature, other populations displayed significantly higher percentages: 22.8% in Turkish [30], 18.9% in Jordanians [29], 16% in Egyptians [32], and 6% in Iranians [31].

### 4.4. C‐Shaped Canal

In this study, the occurrence of C‐shaped canals in the mandibular first and second molars was 0.30% and 4.5%, respectively, which is significantly lower than reported in previous studies. Earlier studies showed varying prevalence rates of 24.33% for mandibular first molars in a Saudi population [34], 10% in a Sudanese population [27], and 1.2% in an Iranian population [23]. For mandibular second molars, the obtained percentage was significantly lower compared to 30.33% in a Saudi Arabian population [34], 12.8% in Egyptians, 10.4% in Jordanians [29], 10% in Sudanese [27], and 3% in Iranians [31]. Studies have shown that the incidence of C‐shaped canals is higher in mandibular second molars, which is consistent with our outcomes [23, 35, 36]

### 4.5. RE

No cases of RE were identified in the first mandibular molars. In contrast, one case (0.3%) of RE in a male North African patient was noted. Studies have shown RE rates of 21.09% in Taiwanese Chinese [28], 0.85% in Turkish [30], 4% in Jordanians [29], and 4.3% in Saudi Arabians [26]. Additionally, a higher prevalence of RE has been reported in mandibular first molars than in second molars [25, 37], contradicting our findings. If CBCT scans are unavailable, multiple angled radiographs are essential for accurate identification of these teeth, and the access cavity should be modified to a trapezoidal shape to facilitate successful RE canal localization. Based on Carlsen and Alexandersen’s classification, our case was outlined as an AC type.

### 4.6. RP

No cases of RP were observed in the mandibular first or second molars. Similar to RE, the prevalence of RP has been reported to be higher in mandibular first molars than in second molars [37, 38]. Therefore, the access cavity of teeth with RP should generally be wider bucco‐lingually.

### 4.7. MCC

No cases of MMC were reported in this study, whereas the existing literature has shown that MMC prevalence ranges from 1% to 23% among different countries [39]. Moreover, MMC has been detected more frequently in mandibular second molars than in mandibular first molars [40, 41]. Such variability in prevalence may reflect differences in ethnicity, sample size, imaging resolution, and operator experience. It is also possible that MMCs were present but not detected due to methodological limitations.

Clinically, there is a strong anatomical association between MMCs and mesial isthmi suggesting that the presence of an isthmus should raise clinical suspicion for an additional canal [42, 43]. Failure to explore the dentinal protuberance between the mesiobuccal and mesiolingual canals may lead to underdiagnosis. A sharp endodontic explorer (DG 16), a small stiff stainless steel K‐file, or micro‐openers can be used for this purpose [44]. In addition to radiographs and tactile methods, several adjunctive techniques can be used to enhance the detection of additional or atypical root canals. The champagne bubble test, which involves placing sodium hypochlorite in a pulp chamber and observing the release of air bubbles, can help identify canal orifices that are not visible to the naked eye [45, 46]. The methylene blue staining technique is another valuable aid. Applying the dye to the pulp chamber floor can help delineate fine developmental grooves or hidden canal entries [47]. Adequate illumination and thorough exploration of the pulpal floor adequate illumination and thorough exploration of the pulpal floor with the aid of magnification are key elements in identifying various canal configurations, especially when combined with CBCT.

## 5. Limitations

In this study, a wide‐field‐of‐view CBCT scan was used; however, using a limited‐field‐of‐view CBCT could have provided more accurate insights into the specific details of the analyzed root canal systems. This may explain the absence of RE, RP, and particularly MMC in our findings, as these structures could be very fine or narrow and might have been overlooked if not clearly patent or partially calcified. Additionally, technical limitations, such as the large voxel size of 450 μm, low‐contrast resolution, and motion artifacts, may have caused limitations in detecting MMC in CBCT. Comparable studies employing similar settings have successfully detected MMC, making it reasonable to infer that the absence of MMCs in our findings reflects their true absence within our specific sample, rather than incomplete detection due to methodological constraints [48].

Another constraint was the relatively small sample size and the ethnically heterogeneous nature of our study population, which may have inherently exhibited a lower prevalence of these anatomical variations compared with global averages. The observed differences in root canal configurations across populations suggest that ethnic, genetic, and environmental factors play an essential role in these anatomical variations. Exploring the genetic factors underlying them through genome‐wide association studies can provide valuable insights into genetic predispositions, particularly in diverse populations. Furthermore, examining epigenetic factors can reveal how environmental influences, such as nutrition, systemic conditions, or even prenatal factors, affect root and canal development. Further clinical studies should employ innovations in diagnostic technologies, outcome‐based designs, and improvements in education and training to enhance the accurate detection and successful treatment of atypical canal anatomy.

## 6. Conclusion

Mandibular second molars were more prone to anatomical variations than first molars, particularly C‐shaped canal configurations. The majority of permanent mandibular first molars exhibited two roots and four canals, whereas permanent mandibular second molars typically had two roots and three canals. Cases of RE were exclusively observed in a single mandibular second molar, with no instances of RP or MMC identified in our study population.

By contributing new data to the existing literature, this study underscores the importance of thorough knowledge of root canal variations in first and second mandibular molars across different ethnic regions and nationalities. Early radiologic identification allows cautious treatment of endodontic structures and preparedness for the early detection of anomalies, specifically in mandibular second molars and in patients of South Asian and Middle Eastern origin, where most variations were found. The findings also emphasize the importance of modifying traditional treatment approaches, using advanced diagnostic tools, and referring patients to specialists when necessary to ensure comfort, satisfaction, and overall care success.

## Author Contributions

**Shaima Dheyab** and **Entisar AlRasasi**: conceptualization, methodology, validation, formal analysis, investigation, resources, data curation, writing (original draft), writing (review and editing), visualization, project administration. **Ahmed M. Aziz**, **Saad Albayatti**, and **Mehmet Omer Gorduysus**: writing (review and editing), supervision.

## Funding

No funding was received for this study.

## Conflicts of Interest

The authors declare no conflicts of interest.

## Data Availability

The data supporting the findings of this study are available on request from the corresponding author. The data are not publicly available due to privacy or ethical restrictions.
